# Study on the Performance of Aniline Electrodeposited on MnO_2_ Nanowire as an Anode for Sodium-Ion Batteries

**DOI:** 10.3390/polym16131856

**Published:** 2024-06-28

**Authors:** Dandan Ma, Xiangyu Yin, Xinyi Li, Xiangge Qin, Meili Qi

**Affiliations:** 1 School of Materials Science and Engineering, Jiamusi University, Jiamusi 154007, China; madandan@jmsu.edu.cn (D.M.); 18863017513@163.com (X.Y.); 2 School of Information Science and Electronic Technology, Jiamusi University, Jiamusi 154007, China; 3 School of Pharmacy, Jiamusi University, Jiamusi 154007, China; lixy0128@foxmail.com

**Keywords:** sodium-ion battery, nanowires, MnO_2_, PANI, anode

## Abstract

Manganese dioxide is an ideal anode for sodium-ion batteries due to its rich crystal shapes. However, its low conductivity, low reversible discharge capacity, slow diffusion kinetics, and poor cyclic stability limit its potential for industrial application. The design of manganese dioxide (MnO_2_) with various morphologies, such as nanowires, nanorods, and nanoflowers, has proven effective in enhancing its electrochemical performance. Stacking nanowire structures is of interest as they increase the open space by forming an interconnected network, thus facilitating favorable diffusion pathways for sodium ions. Concurrently, the substantial increase in the electrolyte contact area efficiently mitigates the strain induced by the volume expansion associated with the repetitive migration and insertion of sodium ions. Based on previous research, this work presents the structural design of flexible MnO_2_/polyaniline (MnO_2_/PANI) nanowires assembled on carbon cloth (CC), an innovation in MnO_2_ modification. Compared to conventional MnO_2_ nanowires, the MnO_2_/PANI nanowires exhibit enhanced structural stability and improved dynamic performance, thereby marking a significant advancement in their material properties. This MnO_2_/PANI composite exhibits a rate capacity of approximately 200 mA h g^−1^ after 60 cycles at a current density of 0.1 A g^−1^, and maintains a rate capacity of 182 mA h g^−1^ even after 200 cycles under the same current density. This study not only provides new insights into the underlying mechanisms governing energy storage in MnO_2_/PANI nanowires but also paves the way for their further development and optimization as anodes for sodium-ion batteries, thereby opening up fresh avenues for research and application.

## 1. Introduction

In recent years, with the rapid development of the new energy automobile industry and large-scale electrochemical energy storage systems, the demand for lithium resources continues to increase and has begun to show a supply shortage [1,2]. Compared with lithium resources, our country has a relatively rich distribution of sodium resources and a more reliable supply of raw materials. Sodium-ion battery technology has high safety, low cost, abundant reserves, and good temperature adaptability and is expected to become an important supplement for the application of secondary batteries [2,3,4,5]. Although sodium and lithium belong to the same main group of elements with similar characteristics, lithium cannot be simply and directly replaced with sodium because sodium has a larger ion size and slightly different chemical properties. This makes sodium-ion batteries have low energy density, high internal resistance, and unstable electrode material, resulting in increased energy loss and declined performance during the charge and discharge process [6,7,8,9,10]. Therefore, it is an important challenge to find suitable anode for sodium-ion batteries.

Among various types of materials, transition metal oxides are currently the most popular anode. The transitional oxide material has a large S-shaped channel and a small hexagonal channel, and sodium ions can rapidly diffuse and have good structural stability, thus showing considerable specific discharge capacity and excellent cycling performance [8,11]. Transition metal oxides are mainly manganese, iron, cobalt, nickel, or copper oxides; among these, manganese oxide is a great potential material. Their catalytic activity, adsorption capacity, stability, and other properties can be adjusted by changing the crystal shape, morphology, and specific surface area [12,13,14,15]. Nor Fazila Mahamad Yusof et al. investigated Mn_2_O_3_ powders prepared by the MnCO_3_ thermal conversion method, and their application as a sodium-ion battery anode was reported for the first time. The cubic grain size of Mn_2_O_3_ is about 1.0 to 1.5 µm, and the Mn_2_O_3_ subunits formed on its surface contribute to the disinsertion/insertion of sodium ions. The initial discharge capacity of Mn_2_O_3_ is 544 mA h g^−1^, and its capacity remains at 85% after 200 cycles at 100 mA g^−1^ [16]. Zhang et al. synthesized nanostructured manganese dioxide (nanorods and nanoflower) by a simple two-step hydrothermal method and heat treatment and studied it as an anode for SIBs. At a current density of 50 mA g^−1^, the initial sodium-ion storage capacity of MnO_2_ nanorods and nanoflowers was 427.4 and 487.8 mA h g^−1^, respectively. In particular, MnO_2_ nanoflowers showed good rate performance (103.3 mA h^−1^ at 800 mA g^−1^ after 100 cycles) and satisfactory cyclicity (133.6 mA h g^−1^ at 400 mA g^−1^ after 1000 cycles) [17]. Van Hoang Nguyen et al. reported an electrode material using Ni-doped layered manganese dioxide for sodium-ion batteries. In Ni-doped layered MnO_2_ (0.05–0.15wt%) prepared by the sol-gel method using fumaric acid as the chelating agent, they showed that layer spacing gradually increased with the increase in the Ni doping amount. In the charge and discharge test, the initial capacity of 15% Ni layered MnO_2_ was 140 mA h g^−1^, and the capacity attenuation was small after 20 cycles [18]. Different nanostructures of manganese oxide are used in sodium-ion battery applications, but so far there are no reports on the study of MnO_2_ nanowires as anode for sodium-ion batteries [17,18,19,20].

In this paper, the MnO_2_/PANI nanowire structure is designed and studied as a sodium-ion battery anode for the first time. By electrodepositing a thin layer of PANI on the MnO_2_ nanowires, the conductivity is increased and the electron transport distance is shortened, thus ensuring the reversible electrode reaction [21,22,23]. In order to verify the advantages of the design of MnO_2_/PANI nanowire structures, the electrochemical performance of MnO_2_/PANI nanowire is characterized in this paper, and the sodium storage mechanism of MnO_2_/PANI nanowire is studied by investigating the relationship between structure and property.

## 2. Experimental 

### 2.1. Preparation of MnO_2_ Nanowires on Carbon Cloth

MnO_2_ was prepared on carbon cloth by hydrothermal method. Firstly, KMnO_4_ was dissolved in 35 mL deionized water (DI), magnetically stirred for 20 min, and then 0.875 mL concentrated hydrochloric acid was added, stirred for 20 min, and transferred to a Teflon-lined stainless steel reactor. A piece of carbon cloth was put into an autoclave containing the mixed solution. Then, the autoclave was placed into a drying oven and heated at 160 °C for 12 h. After cooling to room temperature, the carbon cloth was washed and dried to obtain MnO_2_ on the carbon cloth. Then, the MnO_2_ nanowire on carbon cloth was obtained by placing the MnO_2_ carbon cloth into a tubular container and heat-treating it in 400 °C air for 2 h.

### 2.2. Preparation of MnO_2_/PANI Nanowires 

The electrolyte solution was prepared with 100 mL deionized water, 1 mL H_2_SO_4,_ and 1mg aniline and thoroughly stirred. The MnO_2_ carbon cloth acted as the research electrode and the carbon rod as the reference electrode. When the current was 0.03 A, the potential was 10 V, and the deposition time was 30 s, a thin layer of polyaniline was deposited on the MnO_2_ carbon cloth. MnO_2_/PANI nanowires were obtained by removing the remaining electrolytes and other impurities with deionized water.

### 2.3. Materials Characterization

The morphology of the samples was studied with a scanning electron microscope (SEM, JSM-6360LV, JEOL, Tokyo, Japan) and a transmission electron microscope (TEM, Fei Tecnai G2 F20 S-Twin [AGB1], Bellaterra, Spain), and high-resolution TEM (HRTEM) images were collected. The crystal structure was analyzed by X-ray diffractometry (XRD, D8Advance, Bruker AXS, Billerica, MA, USA). Fourier transform infrared (FTIR) spectra were used to investigate the structure changes of polyaniline after electrodeposition. 

### 2.4. Electrochemical Measurements

The MnO_2_ nanowires and MnO_2_/PANI nanowires were cut into squares with 8mm sides as anodes. The test coin cells were assembled in a glove box filled with argon. NaPF_6_ was used as the electrolyte. A Na sheet and glass fiber were used as the cathode and separator, respectively. A CT2001A LAND battery test system was used to measure the galvanostatic charge/discharge (GCD) in the voltage range of 0.01–3 V. A CHI 660E electrochemical workstation was used to test cyclic voltammetry (CV) from 0.01 to 3.0 V (vs. Na^+^/Na) at a scan rate of 0.1 mV s^−1^. The electrochemical impedance spectroscopy (EIS) was measured by sweeping the frequency from 0.01 Hz to 100 kHz. The capacity of the MnO_2_/PANI electrode was calculated based on the total mass of MnO_2_ and PANI. The load capacity of MnO_2_ was about 1.67~2.32 mg cm^−2^. 

## 3. Results and Discussion

### 3.1. Morphology and Structure

**Figure 1:**
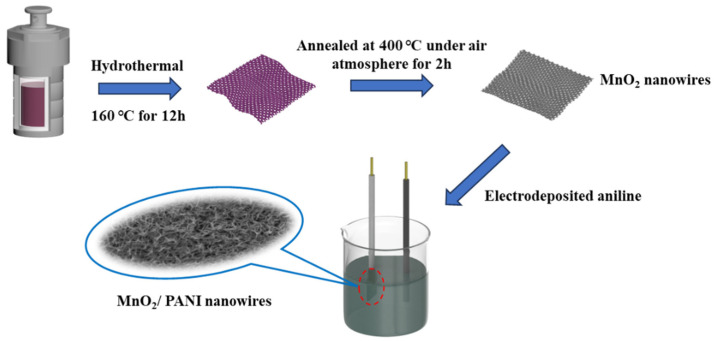
Schematic of the synthesis procedure of the MnO_2_/PANI nanowires.

The synthesis procedure of the MnO_2_/PANI nanowire is shown in Figure 1. A suitable temperature and time are key factors for the synthesis of MnO_2_/PANI nanowire. 

**Figure 2:**
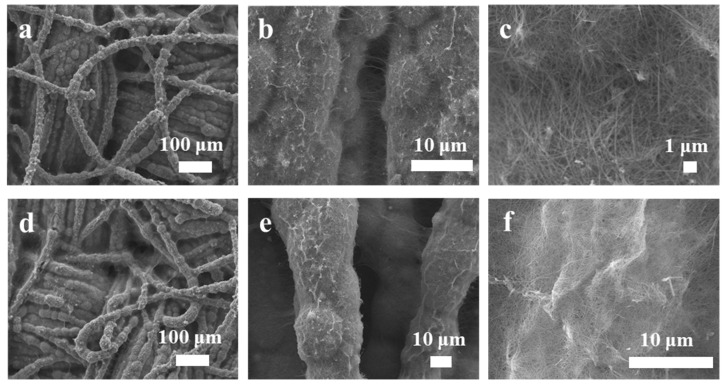
SEM images of CC: (**a**–**c**) MnO_2_ nanowires, (**d**–**f**) MnO_2_/PANI nanowires.

In order to illustrate the synthesis and optimization of MnO_2_/PANI nanowire structures, the morphologies of the MnO_2_ nanowires and MnO_2_/PANI nanowires were characterized using a scanning electron microscope (SEM). Figure 2a,c shows the obtained SEM images of MnO_2_ nanowires. MnO_2_ nanowires are grown uniformly on CC. The surface of CC becomes rough and is evenly covered with hundreds of nanometers of MnO_2_ nanowires in Figure 2b. Figure 2c shows the enlarged SEM image, where MnO_2_ on CC is a typical nanowire structure. Figure 2d,f shows the SEM image of MnO_2_/PANI obtained on CC. Compared to MnO_2_ nanowires, the surface of CC becomes rougher. Figure 2f shows the surface of MnO_2_ nanowires on CC covered with a layer of PANI.

**Figure 3:**
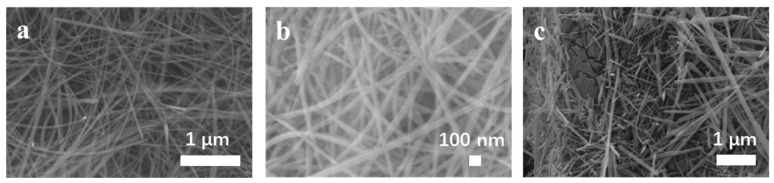
SEM images of CC (**a**) MnO_2_ nanowires, (**b**) MnO_2_/PANI nanowires, and (**c**) aniline electrodeposited over 1 min on MnO_2_ nanowires.

Figure 3a,b shows the morphologies of MnO_2_ nanowires and MnO_2_/PANI nanowires more clearly. Figure 3a shows that the MnO_2_ nanowires are composed of typical one-dimensional nanowires with a diameter of 20–50 nm. Figure 3b displays the MnO_2_/PANI nanowires after electrodeposition, which have a diameter of 35–75 nm, are about a few microns in length, and are separated well. Before electrodeposition, the MnO_2_ nanowires are smooth. After electrodeposition, the diameter of the MnO_2_/PANI nanowires increases and the surface becomes rough [24]. Figure 3c verifies that when the electrodeposition time exceeds 60 s, PANI will form clumps on the carbon cloth, and part of the MnO_2_ nanowires will wrap completely, which will inevitably destroy the stability of the structure. Therefore, a 30 s electrodeposition time is more appropriate.

**Figure 4:**
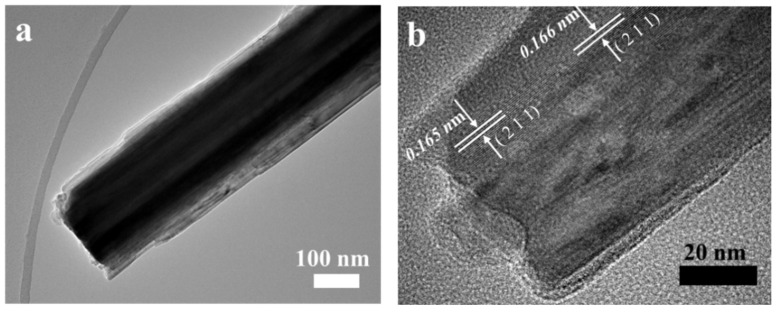
(**a**) TEM image and (**b**) HRTEM image of MnO_2_/PANI nanowires.

The samples require further analysis by TEM and HRTEM. Figure 4a exhibits a classical TEM image of the MnO_2_/PANI nanowires. It can be seen that on the MnO_2_/PANI nanowires with diameters of about 100 nm, the light-colored portion at the edge is coated by PANI, confirming that aniline has been electrodeposited on the MnO_2_ nanowires. The results are consistent with SEM observations, it is again proved that this method synthesizes the material of MnO_2_/PANI nanowires. In Figure 4b, the HRTEM result is displayed. The surface of MnO_2_ nanowires was coated with PANI with a thickness of about 3 nm. HTTEM image of the MnO_2_/PANI nanowires shows the two adjacent lattices spacing are 0.165 nm and 0.166 nm respectively, which is consistent with the spacing of MnO_2_(211) planes. It is proved that the main components of nanomaterials are MnO_2_ and PANI [2]. 

**Figure 5:**
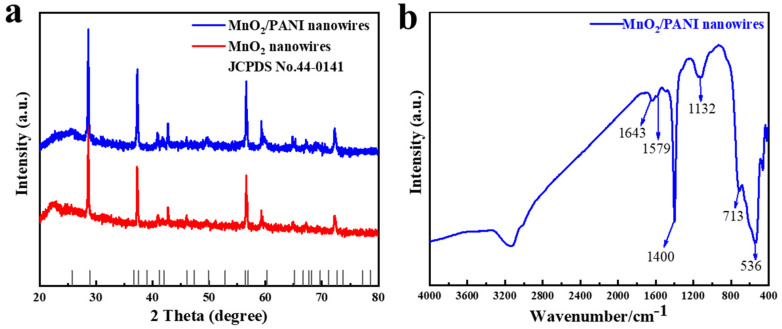
(**a**) XRD patterns of MnO_2_ nanowires and MnO_2_/PANI nanowires; (**b**) FTIR spectrogram of the MnO_2_/PANI nanowires.

The XRD patterns of MnO_2_ nanowires and MnO_2_/PANI nanowires were investigated, as shown in Figure 5a. The diffraction peaks of MnO_2_ nanowires and MnO_2_/PANI nanowires all conformed to the MnO_2_ standard card. The typical diffraction peaks at 28.6°(310), 37.3° (211), 41.8° (301), 49.6° (411), 56.4° (600), 59.3°(521), 64.8°(002), and 72.3°(312) can be determined by the existence of a pure MnO_2_ phase (JCPDS NO. 44-0141) [2]. MnO_2_ nanowires had a peak at 22.4° near the 2 Theta, which was caused by the graphitization of part of the material. MnO_2_/PANI nanowires had a peak at 24.5° near the 2 Theta; this is consistent with the characteristic peak of PANI reported in the related literature. Due to the molecular interaction between PANI and MnO_2_ nanowires, the characteristic peaks of PANI became wider and the crystallinity of the nanowires changed.

Fourier transform infrared (FTIR) studies were used to determine the structure and chemical composition of MnO_2_ nanowires after electrodeposition. As shown in Figure 5b, the characteristic peaks at 1579 and 1643 cm^−1^ come from the C-C stretching vibration of the benzene ring, of which 1579 cm^−1^ is the benzene structure and 1643 cm^−1^ is the quinone structure. The intensity of these two absorption peaks is weak, which can reflect the low oxidation degree of polyaniline. In addition, the infrared absorption peaks at about 536 cm^−1^ and 713 cm^−1^ are attributed to the stretching vibrations of the Mn-O bonds and the Mn-O-Mn bonds in the regular octahedron [MnO_6_]; this further explains the formation of MnO_2_. The other characteristic peaks at 1400 cm^−1^ (C-N stretching vibration) and 1132 cm^−1^ (C-H bending vibration) illustrate the existence of PANI [25,26,27,28]. Therefore, the XRD and FTIR results consistently show that MnO_2_/PANI nanowires were prepared by the electrodeposition of aniline on MnO_2_ nanowires on carbon cloth in this work. 

### 3.2. Electrochemical Performance

**Figure 6:**
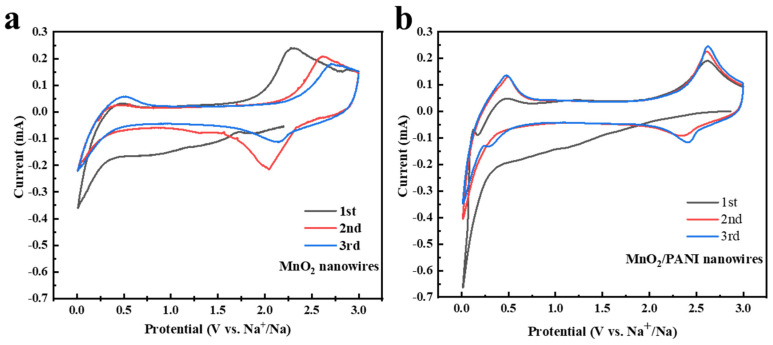
CV curves of (**a**) MnO_2_ nanowires and (**b**) MnO_2_/PANI nanowires at a scan rate of 0.1 mV s^−1^ in the potential range of 0.01–3.0 V (vs. Na^+^/Na).

To estimate the excellent electrochemical performance of the MnO_2_/PANI nanowire anode in sodium-ion batteries, a coin cell was assembled. Cyclic voltammetry (CV) curves of MnO_2_ nanowires and MnO_2_/PANI nanowires are shown in Figure 6. By comparing Figure 6a,b, we can see that in the first cycle, the irreversible reduction peak appears in both graphs, corresponding to the formation of an SEI film. Since the second cycle, the peak no longer appeared, indicating that the formed SEI film was stable. After the activation of the first cathode scanning, the anode peaks of the second and third cycles completely coincided with subsequent anode–cathode scanning (Figure 6b). The peak position and peak area remained stable, indicating that the MnO_2_/PANI nanowire anode features a more stable cycle, high reversibility, and no attenuation of specific discharge capacity compared to MnO_2_ nanowires. A couple of redox peaks can be observed for MnO_2_/PANI nanowires at around 2.6 V/2.4 V and MnO_2_ nanowires at around 2.6 V/2.0 V, illustrating the migration and insertion of sodium ions. In Figure 6b, the potential value between the oxidation peak and the reduction peak is very small (0.2 V), indicating that the polarization is weak, while the potential difference in Figure 6a is large (0.6 V), indicating that the polarization is more obvious, which will accelerate the consumption of electrode materials and waste energy. In addition, the peak intensity of MnO_2_/PANI nanowires is larger than that of MnO_2_ nanowires, showing that the electrodeposition of polyaniline has better conductivity [2,9].

**Figure 7:**
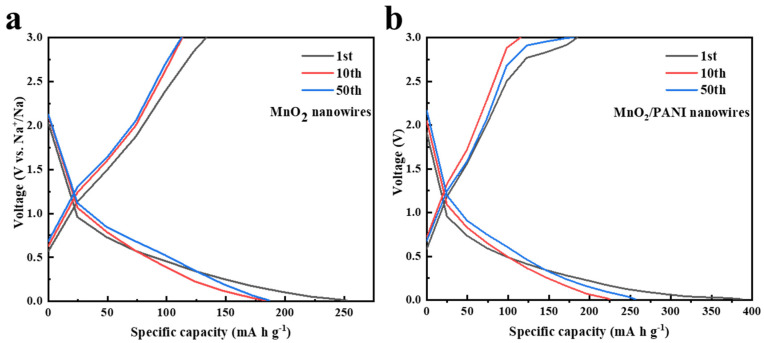
Galvanostatic discharge/charge curves in selected cycles for (**a**) MnO_2_ nanowires and (**b**) MnO_2_/PANI nanowires at a current density of 100 mA g^−1^.

As shown in Figure 7, it can clearly be seen from the galvanostatic discharge/charge curves under 0~3V at the low current density of 100 mA g^−1^ that the charge/discharge capacity of MnO_2_/PANI nanowires in the 1st, 10th, and 20th cycles was higher than that of MnO_2_ nanowires. The first discharge and charge capacities of MnO_2_ nanowires are 252 mA h g^−1^ and 133.5 mA h g^−1^, with an initial coulomb efficiency of 53%, while the first discharge and charge capacities of MnO_2_/PANI nanowires are 387 mA h g^−1^ and 185 mA h g^−1^, with an initial coulomb efficiency of 47.8%. The loss of capacities in the initial cycle may be related to the formation of SEI and to an irreversible reaction on the surface of the material. But in the following cycles, after the electrodeposition of polyaniline, the conductivity of the electrode material increases. The anode modified by PANI in sodium-ion batteries exhibits a higher initial capacity, cycle stability, and rate capability. Reversible capacity 256 mA h g^−1^ at the 5th cycle and 224.7 mA h g^−1^ at the 10th cycle, and there is no obvious discharge platform during the discharge process, which is basically consistent with the results of the CV curves (Figure 6b). This indicates that no phase transition occurred during the charge and discharge process after the electrodeposition of PANI. We suppose that PANI can strongly stabilize the material structure, prevent the phase transition, and make the structure extremely stable, which provides new insights into the open framework for the rapid transport of sodium ions [15].

**Figure 8:**
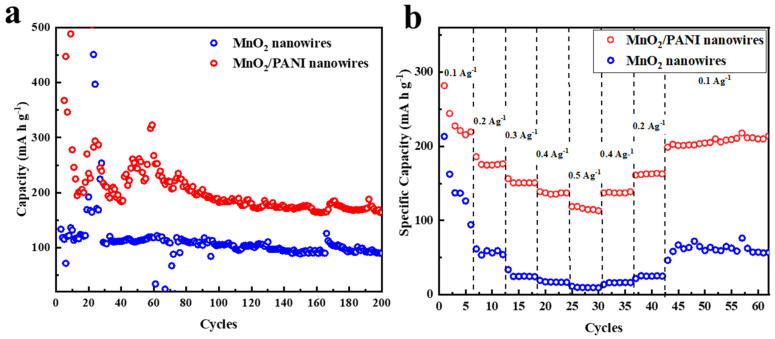
(**a**) Cyclic performances at a current density of 100 mA g^−1^. (**b**) Rate performance at various current rates ranging from 0.1 to 0.5 A g^−1^.

The comparison of cyclic performance between MnO_2_ nanowires and MnO_2_/PANI nanowires is shown in Figure 8a. During the initial 80 cycles, the discharge capacity decayed, and then stayed stable. The discharge capacities of MnO_2_ nanowires and MnO_2_/PANI nanowires are 90.3 mA h g^−1^ and 167.7 mA h g^−1^ after 200 cycles. MnO_2_/PANI nanowires manifest superior cycle ability than MnO_2_ nanowires. In Figure 8b, the MnO_2_/PANI nanowire electrode also shows a better rate performance than the MnO_2_ nanowire electrode. The discharge capacity of MnO_2_/PANI nanowires is 215.2, 174.7, 150.4, 135.2, and 114.4 mA h g^−1^ at current rates of 0.1, 0.2, 0.3, 0.4, and 0.5 A g^−1^, respectively. When the current density is switched back to 0.1 A g^−1^, the specific capacity can still be restored to about 200 mA h g^−1^, which represents more than 95% capacity recovery and shows excellent reversibility and structural stability [2,10].

**Figure 9:**
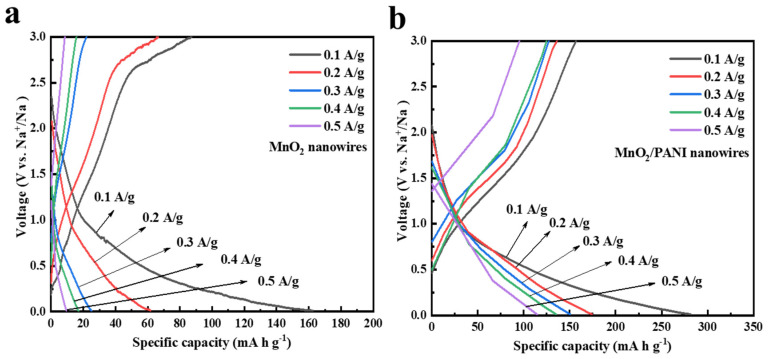
Discharge/charge curves (**a**) MnO_2_ nanowires and (**b**) MnO_2_/PANI nanowires at various current rates ranging from 0.1 to 0.5 A g^−1^.

Further, the rate properties of MnO_2_ nanowires and MnO_2_/PANI nanowires samples were studied step by step from 0.1 to 0.5 A g^−1^. At 0.1 A g^−1^, 0.2 A g^−1^, 0.3 A g^−1^, 0.4 A g^−1^, and 0.5 A g^−1^ current densities, MnO_2_ nanowires had discharge capacities of 162.5, 61.7, 25, 16.9, and 9.5 mA h g^−1^, respectively (Figure 9a). At a reverse current rate of 0.1 A g^−1^, specific capacity returned to a value of 58.8 mA h g^−1^, indicating that the MnO_2_ nanowire capacity decline was serious, and the cycle performance and structural stability are poor. The discharge capacities of MnO_2_/PANI nanowires were 281.6, 174.4, 150.4, 135.2, and 114.4 mA h g^−1^, respectively (Figure 9b), and the specific capacity returned to a value of about 160 mA h g^−1^ at a reverse current rate of 0.1 A g^−1^. Except for the loss of capacities in the initial cycle, the discharge capacities of other current densities were consistent with those of the rate performance image [13]. In contrast, MnO_2_/PANI nanowires had no obvious capacity attenuation trend and had better performance, structural stability, and reversibility.

**Figure 10:**
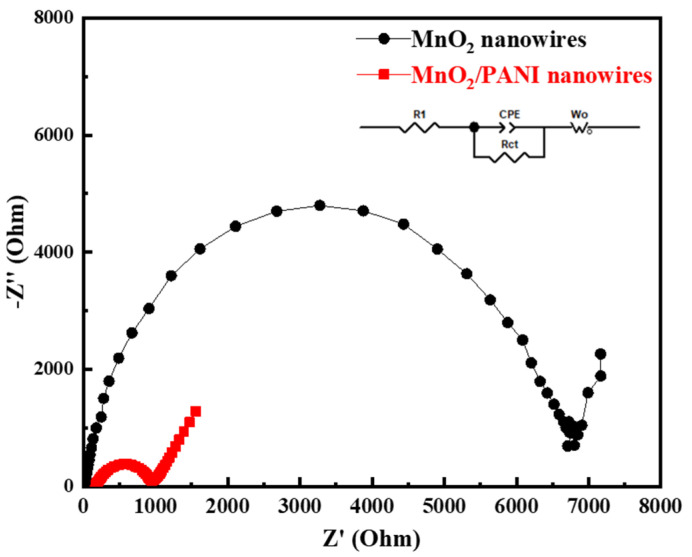
Electrochemical impedance spectra of MnO_2_ nanowires and MnO_2_/PANI nanowires.

To further illustrate the excellent electrochemical performance of MnO_2_/PANI nanowires and the diffusion kinetics of sodium ions, electrochemical impedance spectroscopy (EIS) analysis was conducted on MnO_2_ nanowires and MnO_2_/PANI nanowires. Figure 10 shows the impedance profile in the frequency range between 100 kHz and 0.01 kHz, which usually consists of a semicircle in the high-frequency region and a 45° straight line in the low-frequency region. The equivalent circuit of EIS curves includes R1, Rct, CPE, and W0, where "R1" represents the ohmic resistance encountered by sodium ion when it is transferred through electrolytes and devices, Rct is the interface transfer resistance, and W0 shows the diffusion of sodium ion from the electrolyte to the electrode surface. It can clearly be seen that the semicircular diameter of MnO_2_/PANI nanowires is much smaller than that of MnO_2_ nanowires, indicating that the combination of PANI and MnO_2_ nanowires effectively reduces the interfacial diffusion resistance of MnO_2_ nanowires and improves the interfacial reaction kinetics.

### 3.3. Analysis of Electrode Structure after Circulation

**Figure 11:**
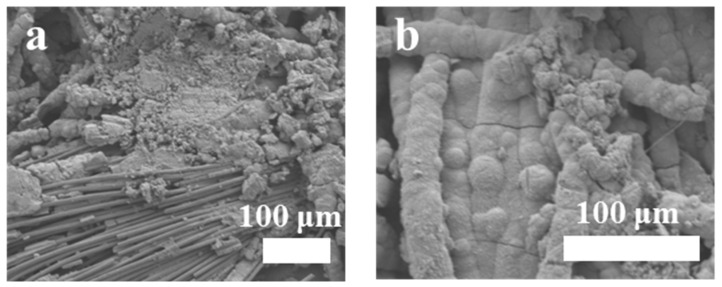
SEM images of (**a**) the MnO_2_ nanowires and (**b**) the MnO_2_/PANI nanowires after 200 cycles.

In order to further confirm the relationship between the nanowire structure and cycle stability, we disassembled the post-cycle battery and analyzed the structure and topography. As shown in Figure 11a, after 200 cycles, the non-electrodeposited MnO_2_ nanowire was pulverized, which affected the performance of the cycle. However, the electrodeposited MnO_2_ nanowire (Figure 11b), because the coating of PANI is not powdery, still maintains the original structure, thus improving the stability of the cycle.

### 3.4. Comparison of MnO_2_/PANI Nanowires with Different Materials

We compared MnO_2_/PANI nanowires with other manganese-based anode materials for sodium-ion batteries in order to intuitively understand the properties of manganese-based materials (Table 1). The MnO_2_/PANI nanowire material has better capacity and cycle performance compared with other manganese-based materials.

## 4. Conclusions

In this paper, flexible MnO_2_/PANI nanowires were prepared on carbon cloth by a simple aniline electrodeposition method as anode electrode materials for sodium-ion batteries. Compared with MnO_2_ nanowires, the improvement in the electrochemical performance of MnO_2_/PANI nanowires is mainly attributed to its network structure morphology, which provides a good diffusion path for sodium ions, and the electronic conductivity is improved by deposing a thin layer of PANI on the outside of MnO_2_ nanowire. Under the condition of 100 mA g^−1^, the initial discharge capacity of the MnO_2_/PANI electrode is 387 mA h g^−1^. From the 80th cycle, the capacity reached about 182 mA h g^−1^, and after 200 cycles, the capacity remained basically the same. To a certain extent, sodium batteries can replace lithium batteries. The preparation of MnO_2_/PANI nanowires provides higher reversible capacity and better cycle stability. The obtained structure promotes electrolyte penetration into MnO_2_/PANI nanowires, providing a good diffusion channel to facilitate the rapid transport of ions. This accelerates the charge transfer within the electrode. Therefore, the result provides new evidence for its application in sodium-ion batteries. 

## Figures and Tables

**Table 1:** Comparison of the electrochemical properties of Mn-based anode materials for sodium-ion batteries.

Mn-Based Anode Materials	Voltage Range (V)	Capacity (mA h g^−1^)	Current Density/Cycles
MnO_2_/PANI (nanowires)	0.00–3.00	182	100 mA g^−1^/100th
MnO/graphene [29]	0.50–3.00	191	50 mA g^−1^/100th
MnO_2_ (nanorods) [2]	0.00–3.00	129.2	50 mA g^−1^/100th
MnO_2_ (nanoflowers) [2]	0.00–3.00	177.1	50 mA g^−1^/100th
Mn_2_O_3_ (powders) [15]	0.00–3.00	130	100 mA g^−1^/200th
Mn_3_O_4_ thin film [29]	0.005-3.00	70	100 mA g^−1^/200th

## Data Availability

Data will be made available on request.
